# Supplementary material for: Identification of Vaginal Microbial Communities Associated with Extreme Cervical Shortening in Pregnant Women
Source: J Clin Med. 2020 Nov 10;9(11):3621. doi: 10.3390/jcm9113621 (PMC7698214; doi:10.3390/jcm9113621)
Supplement: Supplementary file 1 [file jcm-09-03621-s001.pdf]

Journal of Clinical Medicine

Supplementary Materials

Identification of vaginal microbial communities associated with cervical shortening in pregnant women at risk for preterm birth

Monica Di Paola[1,a], Viola Seravalli[2,a], Sara Paccosi[3], Carlotta Linari[2], Astrid Parenti[3], Carlotta De Filippo[4], Michele Tanturli[5], Francesco Vitali[4], Maria Gabriella Torcia[5,*] and Mariarosaria Di Tommaso[2]

Affiliations
[1] Department of Biology, University of Florence, Florence Italy.
[2] Department of Health Sciences, Division of Obstetrics & Gynecology, University of Florence, Italy.
[3] Department of Health Sciences, University of Florence, Italy.
[4] Institute of Agricultural Biology and Biotechnology, National Research Council, Pisa, Italy.
[5] Department of Experimental and Clinical Medicine, University of Florence, Florence, Italy.
[a] These authors have equally contributed.
*Corresponding author: maria.torcia@unifi.it

This file includes:
Supplementary Table 1 and 2
Supplementary Figures 1, 2, 3, 4, 5

Supplementary Tables

Supplementary Table 1 ANCOVA analysis with microbiota as grouping variable and gestational age at sampling as covariate adjustment. Output from R software version 4.0.2 for ANCOVA analysis.

ANCOVA output from R				
**P value for interaction between grouping variable (microbiota) and covariate (gestational age) is 0.185**				
**Anova Table (Type III test)**				
	**SumSq**	**Df**	**Fvalue**	**Pr (>F)**
**(Intercept)**	90.05	1	2.9833	0.09131
**Factor(microbiota)**	154.25	4	5.1099	0.02891
**Gestational age**	23.35	1	0.7736	0.38400
**Residuals**	1297.98	40		

Supplementary Table 2. PERMANOVA analysis on PCoA ordination of vaginal microbiota samples.

Variable	R^2	p-value
Cervical length	0.05623	0.235
GDM	0.01743	0.544

Supplementary Figures

A

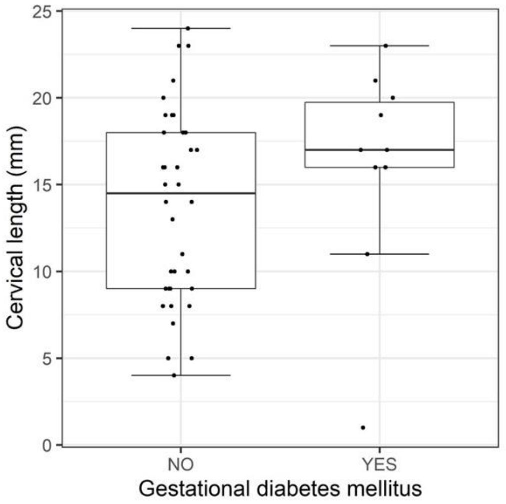

B

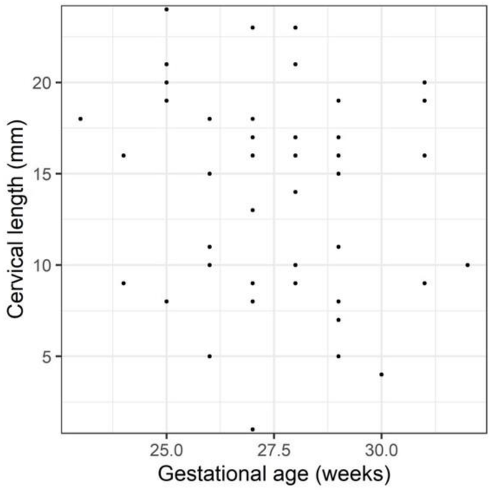

C

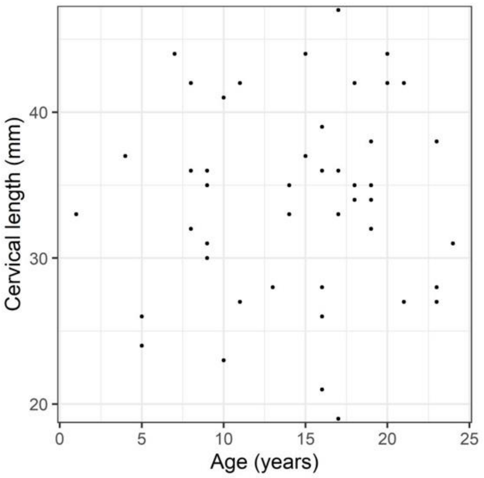

Supplementary Figure 1. A. Differences in cervical length (mm) between patients with or without GDM. Statistical analysis was performed by Mann Whitney test, p value = 0.161. **B.** The relationship between cervical length (mm) and gestational age (weeks) was investigated by Spearman 's rank correlation test. No statistically significant correlation was found (Spearman's rank correlation coefficient -0.108, p value = 0.475). **C.** The relationship between cervical length (mm) and age (years) was investigated by Spearman 's rank correlation test. No statistically significant correlation was found (Spearman's rank correlation coefficient 0.0451, p value = 0.766).

A

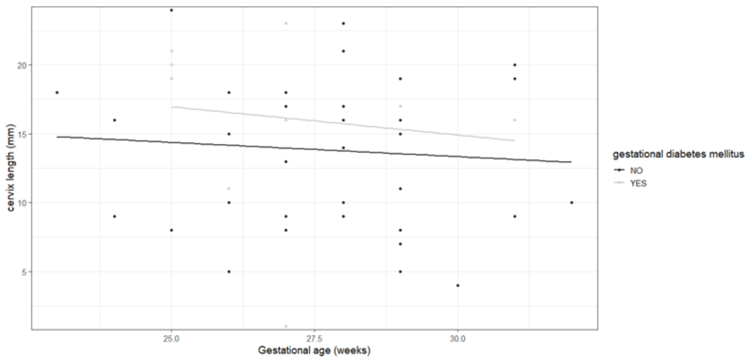

ANCOVA output from R				
P value for interaction between grouping variable (diabetes mellitus) and covariate (age) is 0.851				
**Anova Table (Type III test)**				
	**Sum Sq**	**Df**	**F value**	**Pr (>F)**
**(Intercept)**	102.21	1	3.1011	0.08535
**Factor (gestational diabetes mellitus)**	35.01	1	1.0623	0.30844
**Age**	11.32	1	0.3436	0.56084
**Residuals**	1417.22	43		

B

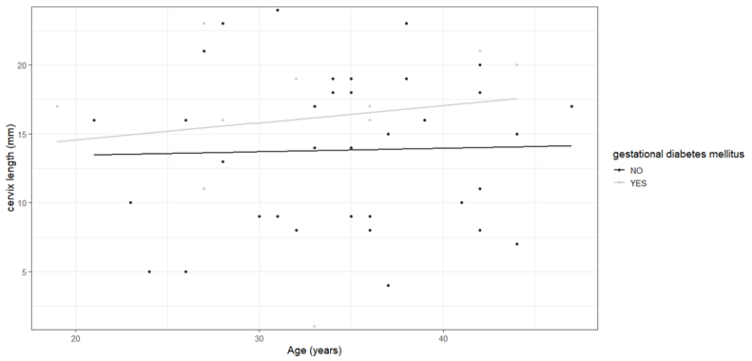

ANCOVA output from R				
P value for interaction between grouping variable (diabetes mellitus) and covariate (age) is 0.741				
**Anova Table (Type III test)**				
	**Sum Sq**	**Df**	**F value**	**Pr (>F)**
**(Intercept)**	229.31	1	6.9273	0.01174
**Factor (gestational diabetes mellitus)**	44.52	1	1.3448	0.25258
**Age**	5.15	1	0.1555	0.69529
**Residuals**	1423.39	43		

Supplementary Figure 2. Subgroups examination and ANCOVA analysis. **A.** The ANCOVA analysis shows that no differences in cervical length between women with or without gestational diabetes mellitus were found when the data were adjusted for gestational age at sampling (p value 0.308). The same analysis shows that no correlation exists between gestational age and cervical length in each subgroup considered. In addition, the ANCOVA analysis revealed that no interaction between the covariate (gestational age at sampling) and grouping variables (gestational diabetes mellitus) exist (p value 0.851). Left panel: scatter plot with regression lines for the two groups (diabetes mellitus yes or no); right panel: output from R software version 4.0.2 for ANCOVA analysis. **B.** The ANCOVA analysis shows that no differences in cervical length between women with or without gestational diabetes mellitus were found when the data were adjusted for gestational age at sampling (p value 0.253). The same analysis shows that no correlation exists between gestational age and cervix length in each subgroup considered. In addition, the ANCOVA analysis revealed that no interaction between the covariate (gestational age at sampling) and grouping variables (gestational diabetes mellitus) exist (p value 0.741). Left panel: scatter plot with regression lines for the two groups (diabetes mellitus yes or no); right panel: output from R software version 4.0.2 for ANCOVA analysis.

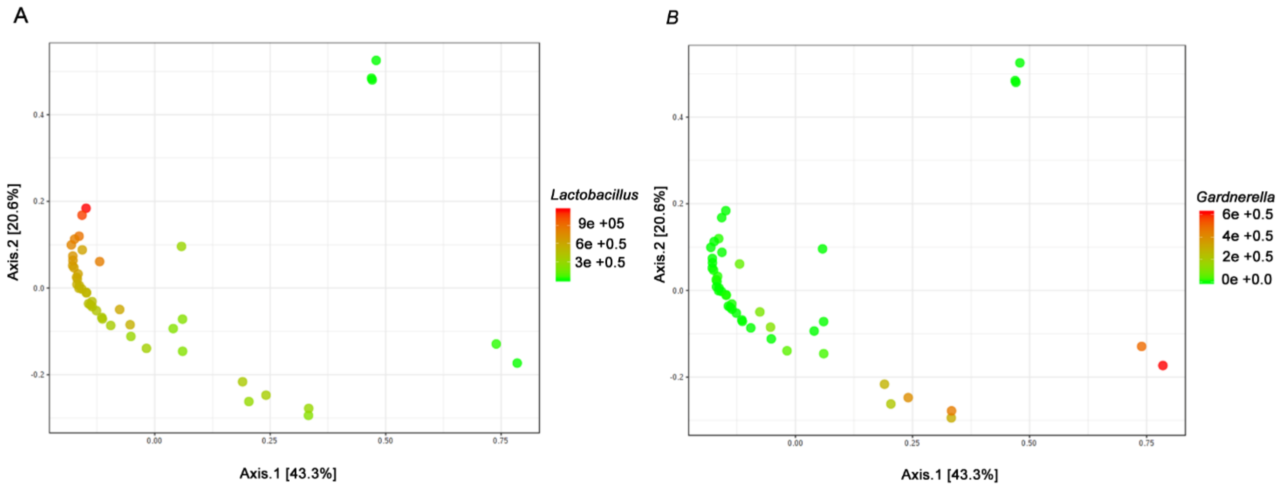

Supplementary Figure 3. PCoA ordination, based on Bray Curtis dissimilarities, correlated with **A.** *Lactobacillus spp.* and **B.** *Gardnerella spp.* abundances. Colors from red to green indicate decreasing abundance of bacterial genus. (PERMANOVA 999 permutations; R20.740 p value< 0.001).

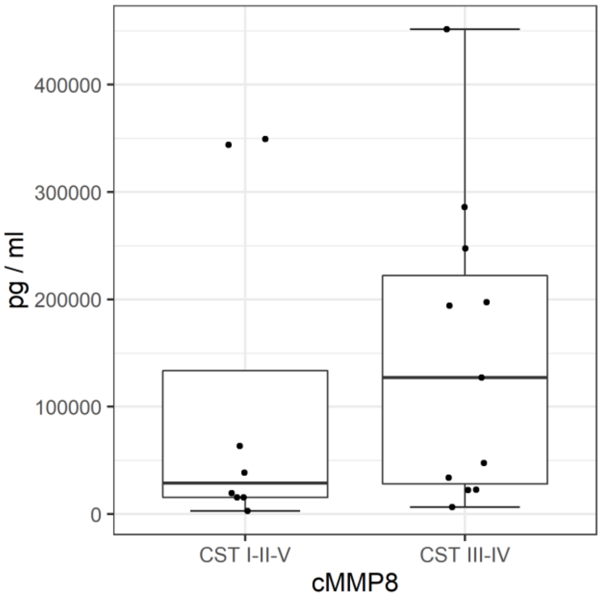

Supplementary Figure 4. Matrix metalloprotease 8 (MMP-8) concentration in vaginal samples of a subgroups of women. Data from pregnant women with vaginal microbiota of CST-I, CST-II and CST-V (*Lactobacillus*- dominated community) were compared with data from women with CST-III (*L. iners*- dominated) and CST-IV (*Lactobacillus*- depleted). Data are presented as box and whisker plots, with boxes extending from the 25th to 75th percentile and horizontal lines representing the median. Whiskers extend 1.5 times the interquartile range from the 25th and 75 th percentile. Statistical analysis, performed by Mann-Whitney assay did not reveal significant differences.

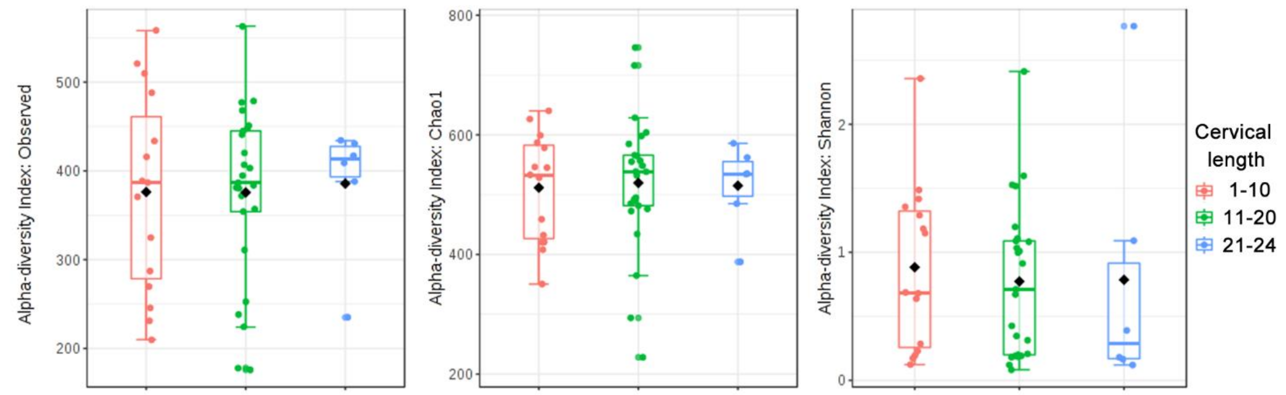

Supplementary Figure 5. Alpha diversity measures. Box plots of observed OTUs, Chao 1, and Shannon index according cervical length classification. ANOVA test resulted not significant for all comparisons.

© 2020 by the authors. Submitted for possible open access publication under the terms and conditions of the Creative Commons Attribution (CC BY) license (http://creativecommons.org/licenses/by/4.0/).
